# The development and tailoring of a peer support program for patients with diabetes mellitus and depression in a primary health care setting in Central Uganda

**DOI:** 10.1186/s12913-020-05301-7

**Published:** 2020-05-19

**Authors:** Dickens Akena, Elialilia S. Okello, Jane Simoni, Glenn Wagner

**Affiliations:** 1 Department of Psychiatry, Makerere University College of Health Sciences, Kampala, Uganda; 2 University of Washington, Los Angeles, USA; 3 RAND Corporation, Seattle, California USA

**Keywords:** Diabetes, Depression, Peer support, Sub-Saharan Africa

## Abstract

**Background:**

About 20–40% of patients with diabetes mellitus (DM) suffer from depressive disorders (DD) during the course of their illness. Despite the high burden of DD among patients with DM, it is rarely identified and adequately treated at the majority of primary health care clinics in sub-Saharan Africa (SSA). The use of peer support to deliver components of mental health care have been suggested in resource constrained SSA, even though its acceptability have not been fully examined.

**Methods:**

We conducted qualitative interviews (QI) to assess the perceptions of DM patients with an experience of suffering from a DD about the acceptability of delivering peer support to patients with comorbid DM and DD. We then trained them to deliver peer support to DM patients who were newly diagnosed with DD. We identified challenges and potential barriers to a successful implementation of peer support, and generated solutions to these barriers.

**Results:**

Participants reported that for one to be a peer, they need to be mature in age, consistently attend the clinics/keep appointments, and not to be suffering from any active physical or co-morbid mental or substance abuse disorder. Participants anticipated that the major barrier to the delivery of peer support would be high attrition rates as a result of the difficulty by DM patients in accessing the health care facility due to financial constraints. A potential solution to this barrier was having peer support sessions coinciding with the return date to hospital. Peers reported that the content of the intervention should mainly be about the fact that DM was a chronic medical condition for which there was need to adhere to lifelong treatment. There was consensus that peer support would be acceptable to the patients.

**Conclusion:**

Our study indicates that a peer support program is an acceptable means of delivering adjunct care to support treatment adherence and management, especially in settings where there are severe staff shortages and psycho-education may not be routinely delivered.

## Background

The prevalence of diabetes mellitus (DM) worldwide stands at 6.7%, and it is anticipated to rise to 7.8% by 2030. A recent population survey in Uganda reported a 7.4% prevalence [1]. Existing evidence shows a rise in the prevalence of DM in sub-Saharan Africa (SSA) [2–4]. About 20–40% of patients with DM, including those from SSA, suffer from depressive disorders (DD) during the course of their illness [5–13]. In our earlier study at three hospitals in Uganda, we found a 35% prevalence of DD among DM patients [14]. Furthermore, DM patients with co-morbid DD are likely to suffer from a number of adversities including sub-optimal hypoglycaemic control, poor medications adherence [15, 16], poor quality of life [17] and increased mortality [18]. A number of efficacious treatments for DD including antidepressants, psychotherapy, or a combination of both [19–21] are available for patients with co-morbid DM, and have been shown to lead to improvement in clinical outcomes including the reduction in DD symptom severity and improvement in glycaemic control [17, 22–29].

Despite the high burden of DD among patients with DM in SSA, DD are rarely identified and adequately treated at the majority of PHC clinics [9, 30]. A number of factors including health care worker shortages and high patient numbers have been sighted as barriers to the integration of DD care in primary health care (PHC) settings in SSA. Health care worker shortages can limit the time taken to deliver comprehensive DD care to patients— care which includes making a correct diagnosis of a DD and providing psycho-education about the disorder and the need for it to be adequately treated using the available options, as well as ensuring patient follow-up.

In resource constrained clinics, peer support has been suggested as one of the methods of delivering adjunct psychosocial care for persons with chronic illnesses including the provision of adherence counselling, alleviating psychological distress, and making referral of patients who need more help [31]. However, studies examining the impact of peer support on DM outcomes among patients with DD, conducted mostly in high income countries and none in SSA, have provided conflicting findings. Some studies have shown benefits [25, 26] such as improvement in glycaemic control and reduction in DD symptom severity, but others have not [27, 28]. A number of factors including limited peer-patient contacts, inability to specifically provide antidepressants [24–30], and the fact that DD care was not provided by formerly depressed patients could explain negative findings.

In this study, we set out to examine the acceptability of providing peer support to DM patients newly diagnosed with DD. We engaged patients who had suffered from a DD, received treatment and were in clinical remission to provide peer support—this was in contrast with previous studies [32–34] in which peer support was provided by individuals who didn’t share experiences of having suffered from a DD illness. The peer support intervention was developed using adaptations of social support models and theories that were developed by Jane M Simoni (one of the authors of this paper) and colleagues for HIV-positive patients with poor adherence to medications [35–37]. Our proposed model (below) was similar in its use of social (peer) support to enhance self-efficacy (including knowledge about DD as a chronic disease for which there is treatment and DM self-care) and improve adherence to medications among individuals with negative affective states (DD).

**The adapted Jane Simoni Model.** 

## Methods

### Study setting

This study was conducted at the DM clinic of Nsambya Hospital in Kampala City, Uganda. Nsambya Hospital is a Faith based Private-Non-For Profit (PNFP) Hospital. Patients pay minimal fees (subsidized by the main funder, the Catholic Church) to access services. It is a tertiary hospital (level 4), with over 500 beds. The DM clinic has over 1500 registered DM patients, 600–1000 of who are actively accessing care (every 2–3 months). On average, 40–60 patients receive DM care at the clinic on each clinic day. The clinic, which is staffed by 3 nurses, a medical officer and a specialist physician, is open one day per week. The nurses provide most of the DM care including eliciting symptoms, providing medication refills, as well as referring complicated cases to the medical officer within the clinic. The medical officer and specialist physician’s roles include managing DM complications, medical co-morbidities (e.g. hypertension) and adjusting hypoglycaemic medications.

### Access to diabetes care

The majority of patients with diabetes in Uganda access care at the government funded facilities within the city. There are more government than private health care facilities in the city and region. However, medication stock outs and shortage of health care workers are huge barriers to the provision of quality health care in Government funded facilities. Patients who can afford to pay for the services choose the private hospitals, which may not be in the vicinity in which they live.

### Overview of study methods

This study was conducted in three phases. In the first phase, we routinely screened clinic attendees for depression, identified those with major DD (MDD), and initiated them on antidepressant treatment. In the second phase, we approached these treated patients in clinical depression remission, and conducted qualitative interviews (QI) to assess their perceptions about the acceptability of training peers to provide support for patients with comorbid DM and depression. In the third phase, we trained patients who were in clinical depression remission, and had volunteered to be a peer support “buddy” to DM patients who were newly diagnosed with MDD, to implement the peer support intervention.

#### Phase I

##### Identification of patients with MDD for inclusion in the acceptability study

In the first four weeks of the study, trained research assistants (RA) screened all clinic attendees for DD using the short version of the patient health questionnaire [38], the PHQ-2 at triage.

The RA obtained written informed consent from the screen positive cases (PHQ-2 ≥ 3), and then sent them to the nurses for a second round of screening using the PHQ-9. All participants aged 18 years and above with a confirmed diagnosis of DM were eligible for recruitment. Participants would be excluded if they were suffering from an illness for which urgent medical attention was needed.

Screen positive (PHQ-9 ≥ 10) cases were then referred to the medical officer/specialist physician for a MDD diagnostic interview using the Mini International Neuropsychiatric Interview (MINI) [39]. This process was repeated till 10 patients with MDD were identified, agreed to depression treatment and were consented to participate in the study. The participants were initiated on either Amitriptyline 75 mg/day or Fluoxetine 20 mg/day. Participants were re-assessed for MDD symptoms at weeks 4 and 6 using the Hamilton Depression Rating scale (HAM-D) [40].

#### Phase II

##### Assessing the acceptability of conducting peer support for patients with DM

Participants in clinical remission (HAM-D ≤ 17), the medical officer, specialist physician and clinic nurses were all approached by the RA and asked to participate in a qualitative interview after providing written informed consent. The interviews were based on a guide that was developed after collating information from the multiple models of social and peer support [35–37], with questions about participant’s perceptions regarding the acceptability of engaging experienced DM patients to provide peer support to patients newly diagnosed with MDD. Questions focused on the content of a peer support session (do’s and don’ts), the eligibility criteria of a buddy, the process of conducting the sessions (location, number of peers per group), and the number of sessions to be provided by the buddy. The guide allowed for comparisons across individuals and categories of respondents. Questions were open-ended to allow not only the exploration of new leads but also generation of rich narratives.

### Conduct of the interviews

The interviews were conducted by two masters level Social Scientists, [one male and one female] with extensive experience in the conduct of qualitative studies. Prior to starting fieldwork the research assistants received additional two days training in qualitative research methods. All the interviews audio recorded, transcribed and translated into English before the analysis. The summary of the interview guides and probes are available as supplement material to this publication.

All interviews were digitally recorded and transcribed verbatim (regardless of whether the interview was administered in English or Luganda). The interview transcripts were entered into text management software— Atlas.ti. Data were explored to identify key themes and relationships between themes. Notes that referred to basic themes (acceptability, barriers to peer support as well as suggestions to address barriers) were coded. Data segments corresponding to each major theme were retrieved using Atlas.ti. Different memos on different data segments identified were written.

### Analysis plans

We used content analysis (Graneheim & Lundman 2004 [41]) approach to analyse data. The second author read through all the transcripts while listening to the audio recordings to clarify data source, get familiarized with the data and confirm completeness of transcription and accuracy of translation. Then the second author read a sample of transcript and developed codes, which were discussed with the first author before coding the data. Data segments that referred to basic themes (acceptability, barriers to peer support as well as suggestions to address barriers) were coded. The second author conducted the analysis but had an ongoing discussion with the first author and together they reviewed, discussed, and refined codes and analysis outputs. There were no significant disagreements between the two.

#### Phase III

##### Training of buddies and implementation of the peer support model

Information from the first two phases of the study was summarized and included in a peer support manual consisting of information about the eligibility criteria for one to be a buddy, the content of peer support, the number of sessions to provide and the regulations that guide those sessions. Ten patients with treated MDD who were in clinical remission were trained over a 2-day period by the lead author using the adapted peer support model. The workshop included didactic content and role-plays. The manual was translated into the Luganda language, so that participants could refer back to it during implementation of the support sessions. Buddies were taught to strive toward the following goals with their assigned peers: (a) explain the purpose of the contacts as educational and supportive, and aimed at improving adherence to prescribed medications; (b) encourage peers to follow treatment regimens; (c) allow the peer to express worry, anxiety, and concerns so these can be dealt with by the group or Health care workers; and (d) and refer frequently to themselves and their success with their own regimen in order to promote self-efficacy and confidence on the part of the peer recipients. The peers and buddies were encouraged to use the above goals to set achievable targets for each meeting. After the two-day training, ‘graduated’ buddies met in a group for supervision with the lead author to discuss anything that needed clarity before they could be assigned patients newly diagnosed with DD.

### Ethical approvals and informed consent

All participants provided written informed consent. For those who were not able read or write, RA read them the consent forms, and explained the contents of the form. Illiterate individuals (those who could neither read nor write) would then use their thumbprints to sign on the consent forms. We used an inkpad to achieve this. Ethical clearance was obtained from the Makerere University, School of Medicine ethical review committee (SOMREC, Rec **Ref# 2016–109**) and Uganda National Council of Science and Technology (UNCST, **HS20ES**). Institutional permission to conduct the study was obtained from the St Francis of St Raphael Nsambya Hospital ethical committee. These approvals included the use of thumbprints as a means of providing informed consent for those who could neither read nor write.

## Results

### Phase I: participant demographics

There were 6 female and 4 male DM (types I and II) patients with a mean age of 40 years (SD ± 5.5). The mean duration of DM among the patients was 6 years (SD ± 2.8). All the 10 patients who received antidepressants were in clinical remission by week 6 (defined as a HAM-D score ≤ 17). Five participants received 20 mg of Fluoxetine daily and 3 received 75 mg of Amitriptyline.

### Phase II: characteristics of potential buddies

Guiding questions/probes from the Jane Simoni Model were posed to elicit information about what they would perceive as characteristics of potential buddies. These factors were identified in earlier studies conducted by Simoni et al. [36, 37] as mediators of the relationship between social support and clinical outcomes. Generally, participants reported that for one to be a buddy, they need to be mature in age, and lead by example with regards to consistently attending the clinics and keeping appointments, and not to be suffering from any active physical or co-morbid mental or substance abuse disorder.

*‘Age doesn’t matter so much as long as one can articulate issues well. But also, you want someone who will be respected by those older than them. Our clinic has children, you can’t expect a 50 year old man to listen to a 15 year old girl. As long as the person is knowledgeable about what they are saying, people will listen to them, but they must be of age. For example, one needs to be able to speak and write both English and Luganda (the language commonly spoken at the site) since most people here are Baganda (the predominant tribe at the study site). Furthermore, the person needs to show a good example to the others by being neat/clean, patient, and attending clinics regularly. Some of our colleagues are very impatient, they want to go ahead of their fellow patients in the queue, thinking our problems are less than theirs; such people will not be fit to be buddies. Moreover, one doesn’t need to be using things that make them drunk and a nuisance; such people will lose the respect of the patients they are dealing with’.* This response from a 40-50 year old male participant was similar to the descriptions by four other participants, and seemed to reflect their perceptions

### Potential barriers and solutions to delivering peer support

Participants anticipated that the major barrier to the delivery of peer support would be high peer and buddy attrition rates as a result of the difficulty in accessing the health care facility due to financial constraints—it was anticipated that the lack of funds to travel to the clinic (especially for those coming from far away rural areas) would make it difficult for patients to regularly attend the sessions. ‘*You see, I spend over 70,000/= (~20USD) to come here. I can only do that once a month. Many of my colleagues insist on getting medications for two months, so that they save the cost of transport. Now you can see that it will be difficult for people to come here on a weekly basis, especially those from far distances’* said a 30–40 year old female participant.

One of the solutions to this barrier was providing a modest transport refund to participants, and having two peer support sessions a month for the first two months — one of those sessions would coincide with the clinic days, hence there would be no need to reimburse transport costs to the participants on that day. From the third month onwards, the peer support sessions would take place monthly, and would be made to coincide with regular clinic visits. This is reflected in the response of a 30–35 year old female participant, ‘*Maybe you give the attendees some transport refund every time they come here, but remember that some of them may just come to get money and not attend sessions. Also, in government facilities, such services of giving transport refunds may not be sustainable. People are poor in this country. I think the easiest solution would be having these meetings take place twice month, and make it coincide with the clinic day so that people see another reason for coming here’*.

Peer support sessions would have to be scheduled on the days that the patients return for their review to the clinic in order to circumvent some of the barriers to accessing the clinic. The option of having peer support delivered in community was considered cumbersome due to the logistical challenges of support to supervise the process. ‘*In the community, people may start to point fingers at those attending the sessions and stigmatize them for no reasons. They will say those people are sick, look at them. I don’t think many people will be willing to attend the sessions in the community setting’* said a 35–45 year old female participant. Of particular concern was the laxity of other patients to return for clinic appointments, and the buddies reiterated the need to emphasize the importance of routinely return to the clinic to access medical care. ‘*From what I have seen, people with depression need more care than those without, your Doctor needs to talk to you regularly. I would like my colleagues to return to the clinic more often than they usually do,’* said the same 35–45 year old female participant.

### The contents of peer support

In a randomized trial by Simoni et al.(2007) [42], buddies provided informational social support, ways of over-coming poor adherence, and how to deal with mental health issues. We used this information and designed probes to elicit information about what the buddies should talk about during the sessions, including the chronic nature of DM and the need for it to be medically treated, and the fact that DM is often co-morbid with other ailments including DD. Participants tended to agree that buddies would:

(a) mainly talk to their buddies about the fact that DM was a chronic medical condition for which there was treatment that needs to be taken for life, *‘In the communities where we live, there is a lot of misinformation about DM. Some people say it can be cured using herbs, others say it is caused by witchcraft, and others say if you stop taking sugar then you are cured. In my view, we need to give correct information. We could use the myth cards to do this. We need to tell them to eat well, take care of their feet, exercise regularly and stay positive. Some people think they will die tomorrow because they can no longer eat their favourite food like meat and rice,* said a 40–50 year old male participant.

The myth cards are a set of 16 educational materials consisting of pictures and texts (phrased in a yes/no format) that provide information about the aetiology, complication, and treatment options for DM. The cards which are meant to dispel a number of myths about DM— myths such as DM being a contagious disease, or that it can respond to herbal or traditional remedies nurses show are shown to clinic attendees at triage on every clinic visit. The use of the cards also re-emphasizes the information about DM being a medical condition with multiple complications that responds to prescribed treatments.

(b) that patients who suffer from DM are prone to other medical adversities including problems with their sight, feet, kidneys, heart, and virtually all body organs, including psychiatric illnesses like DD which further complicates treatment. *‘When I was suffering from depression, my life was terrible; no one understood me, I felt pain but couldn’t describe it. Things only changed when I got to know about this disease. After telling the peers about the myth cards, the second thing that we need to talk about is how painful this depression is; you can’t sleep, you can’t eat even if one cooks meat, you explain this to the Doctor and he gives you painkillers that don’t work. We need to share this experience with others who have the same problem, and tell them that if they take their medicines well and stop worrying, then all will be fine’.* Said a 35–45 year old female participant.

### Number of sessions

In the trial by Simoni et al.(2007) [42], peer support consisted of twice a month meeting, in addition to weekly calls to peers by their buddies. We used this information as a probe to the participants—there were varying opinions about what would be the minimum number of peer support sessions. One of the participants, a 45–55 year old male reported ‘*peer support should be an ongoing procedure as long as one is still accessing care. You see, these problems really never go away, we need to be in constant touch with those who have volunteered to help us. But I also ask myself, whether one would have the time to always come back and be talked too; besides what would they say in the meetings? It is confusing to me’.* Another 40–50 year old female reported *‘once the participants are done with the session (didn’t say how many), there will be need to ‘assess’ them after a while to ascertain whether they were still practicing what they were taught.’* Still, the participant couldn’t come up with a figure about the number of sessions one should undergo. There was need for guidance about this concept during the interviewers by the RA. The consensus was that there would be two sessions per month, followed by monthly sessions from month three to six, and that this would be the time that most participants would be in clinical remission for DD. It was further agreed through consensus that the sessions would take place under a near-by shade within the hospital compound on the clinic day after the patients had been seen by the Health care workers.

### Phase III: implementation of the model

The peer support model was implemented 4 weeks after buddies had received training. All adult DM patients who presented to the clinic were screened for MDD by trained RA using the PHQ-2 and initiated on treatment as described in the earlier section about identification of patients for recruitment in first phase of the study (above). All patients identified as depressed received antidepressant treatment. After initiation of the patients into depression care, the RA obtained written informed consent from them to participate in the peer support study. The peer support session were delivered by one peer to 5 participants. The number chosen was based on the results from phase I above. The aims of the sessions were to provide information about DM, and DM self-care, improve adherence to medications and return to the facility. Each session lasted 45–60 min and was interactive. Peers were allowed to allay their concerns and fears during the sessions. In the event that medical questions were asked, peers would be guided to report these to their medical practitioners. There were 6 number of sessions and they were meant to achieve the outputs identified in phase I above.

## Discussion

Our study indicates that a peer support program is an acceptable means of delivering adjunct care to support treatment adherence and management, especially in SSA settings where there are severe staff shortages [43, 44]. Staff shortages make it difficult for one to screen for, diagnose, initiate on treatment and provide psycho-education to individuals with mental illness including depression. Moreover, the low levels of mental health literacy [45, 46], high stigma levels [47, 48] and negative explanatory models of mental illness in SSA [49–51] are significant barriers to treatment uptake and adherence to treatment. And yet, depressed patients need to adhere to prescribed treatment over long period of time in order for them to achieve symptom remission and attain adequate social and occupational functioning [52, 53]. Peer support interventions are strategically poised to improve treatment uptake and adherence.

These study findings are in keeping with studies in SSA [42–45] that demonstrated that training lay health care workers is an acceptable method of delivering some components of mental health care. Even though little had been done in the field of training patients with DM to deliver MDD care, this study adds to the current body of knowledge regarding the delivery of mental health care. Similar task-shifted approaches for mental health care have been shown to be efficacious in SSA settings including in Uganda [54–57].

Our study provides a stepping stone for researchers to conduct more detailed longitudinal assessment of peer support in resource constrained settings. More work will be needed to examine the long-term impact of the intervention on treatment outcomes. The identified barriers (distance to the facility) will need to be circumvented for this intervention to yield the intended benefits.

### Limitations

This study was not without limitations. First, the study design makes it difficult to generalize these findings to a larger sample. Secondly, this work was conducted in a private hospital that is better run compared to resource constrained government facilities yet, the majority of patients with DM access government facilities in Uganda. Generalization of these findings to government facilities may be inappropriate. Lastly, this study focussed on a small pilot whose aim was to examine acceptability of peer support and not necessarily long term outcomes.

**In conclusion**, despite the limitations, peer support can be delivered to individuals with co-morbid MDD and DM within a private hospital. More work is needed to examine the acceptability of such an intervention in a government public facility.

## Supplementary information

**Additional file 1.** Interview guide for patients.

## Data Availability

The data from this work will be available for use upon request from the corresponding author (whose email is available in the publication).

## Abbreviations

DD: Depressive Disorders
DM: Diabetes Mellitus
HAM-D: Hamilton Depression Rating scale
LMIC: Low and Middle Income Countries
MDD: Major Depressive Disorder
PHC: Primary health care
PHQ: Patient Health Questionnaire
SOMREC: School of Medicine ethical review committee
QI: Qualitative Interviews
RA: Research Assistant
SSA: Sub-Saharan Africa
UNCST: Uganda National Council of Science and Technology
