# Supplementary material for: The development and tailoring of a peer support program for patients with diabetes mellitus and depression in a primary health care setting in Central Uganda
Source: BMC Health Serv Res. 2020 May 19;20:436. doi: 10.1186/s12913-020-05301-7 (PMC7236139; doi:10.1186/s12913-020-05301-7)
Supplement: Supplementary file 1 — Additional file 1. Interview guide for patients. [file 12913_2020_5301_MOESM1_ESM.docx]

**INTERVIEW GUIDE FOR PATIENTS**

***Introduction: Some of the patients receiving Diabetes mellitus care may experience depression which may require treatment and support. We are exploring the possibility of training patients who have had depression that has been successfully treated to provide peer support to other patients who may be experiencing similar symptoms. The purpose of discussion is to get your thoughts about feasibility and acceptability of such initiative***

1. How common is it for individuals receiving treatment for ***Diabetes Mellitus*** to experience depression (excessive sadness or lost of interests in pleasurable activities, continuous for more than two weeks)?
2. What do you think causes depression among people with Diabetes Mellitus?
3. What kind of care is usually given to such individuals who experience depression?
4. In your opinion, what kind of care do you think they should receive?
5. What are difficulties do such people face in accessing care for depression?
6. What can be done to improve access for depression care they need among people with Diabetes Mellitus?
7. In what ways are individuals with ***Diabetes Mellitus*** and depression able to support each other? (***Probe about existence of peer support groups, kind of support provided and willingness of patients to be part of such the activities***)
8. What role can patients with ***Diabetes Mellitus*** who have had experience with depression play in helping other patients with ***Diabetes Mellitus*** and experiencing depression? (Probe for how they can be help to access and adhere to treatment for both DM and depression)
9. How feasible is it to promote the peers in supporting patients with ***Diabetes Mellitus*** and depression in the clinic? (***Probe about willingness of patients to take part***)
10. What do you think should be the characteristics of such individuals? (***Probe about characteristics such as age, gender, level of adherence, public relations skills, level of education etc)***
11. In your opinion, what should be the core function of these individuals?
12. What methods of work should these individuals adopt?
13. How best can these groups be supported to meet the needs of patients with ***Diabetes Mellitus*** and depression?

**THE BUDDIES' MODEL**

***In this project we intend to train individuals with Diabetes Mellitus who have had experience with depression as peers to help other individuals who have symptoms of depression. These individuals will be referred to as buddies. The main roles of the buddies will be to support peers to adhere to treatment for both Diabetes Mellitus and depression as well as self care. The buddies will be trained to ensure they gain confidence and proper communication skills. They will also be taught on how to set boundaries of do’s and don’ts in the peer-buddy relationship. They will also be taught about the signs and symptoms of depression. The buddies will be required to refer persons whose symptoms flare-up for further treatment. The buddies will trained to recognize symptoms of suicide and treat suicide with the required urgency. During the sessions, they will find out whether peers have any troubles with adherence to medications for both Diabetes Mellitus and the new diagnosis of depression. They will be required to assure and ensure confidentiality....***

1. What do you think of this proposed model?
2. How willing are you to take part in this program of buddies? (***Probe for if the individual is willing to take part as buddy/peer to provide support to other patients)***
3. Do you think people with ***Diabetes Mellitus*** and experiencing depression will accept help from the buddies (***If Yes, probe for facilitators; If no probe hindrances/barriers)***
4. In your opinion, how acceptable is the proposed model? What would be the main challenges in implementing such model?
5. How can the challenges you mentioned be addressed?
6. If we are going to use the model in the clinic: How should antidepressants be dispensed to patients? (Probe for a) whether the buddies can participate in dispensing antidepressants b) if Yes, why? c) How should it be done; d) if now; why?)
7. How frequent should patients come for reviews? (***Probe for rationale of the suggested frequency)***
8. How far should the participants (patients and peers) be residing from the hospital?
9. How long should one have been in the clinic for them to qualify as peers?
10. Please get information on age, occupation, place of work, date and place of interview and when the patient diagnosed with DM
